# Work stressors, work-related quality of life, psychological capital, and innovative behavior in emergency and critical care nurses: a cross-sectional moderated mediation study

**DOI:** 10.3389/fpsyg.2026.1744086

**Published:** 2026-05-29

**Authors:** Qingsheng Zhao, Lijun Cao, Yu Li, Hong Wang, Lixia Li, Huiying Yang, Changmin Zhang

**Affiliations:** 1 Department of Emergency, Qilu Hospital of Shandong University, Jinan, Shandong, China; 2 Department of Nursing, Qilu Hospital of Shandong University, Jinan, Shandong, China

**Keywords:** cross-sectional study, emergency and critical care nurses, innovative work behavior, psychological capital, work stressors, work-related quality of life

## Abstract

**Background:**

Emergency and critical care nurses face high job demands that may be associated with reduced capacity for innovative behavior. Little is known about how work-related quality of life (WRQoL) and psychological capital together influence innovative behavior under the high-strain conditions of emergency and critical care, especially when considering individual differences in psychological capital profiles. This study examined whether work stressors were associated with innovative work behavior and whether WRQoL statistically mediated this association, while also examining whether psychological capital—assessed both as an overall resource and as latent profiles—moderated these associations in a cross-sectional design.

**Methods:**

Using convenience sampling, 533 nurses from emergency departments and intensive care units in Shandong Province, completed validated Chinese versions of the Nurse Stress Source Scale, the Work-Related Quality of Life Scale, the Psychological Capital Questionnaire, and the Innovative Work Behavior Scale. Pearson correlations were computed. Mediation and moderation analyses were conducted using PROCESS, and latent profile analysis (LPA) of psychological capital was performed in Mplus to identify subgroups; moderation by total psychological capital and by identified profiles was subsequently tested. This study was approved by the Ethics Committee of Qilu Hospital, Shandong University (Approval No. KYLL-202405-001-1).

**Results:**

Work stressors were significantly and negatively correlated with innovative work behavior (*r* = −0.356, *p* < 0.01). WRQoL partially mediated this relationship (indirect effect = −0.186, 95% CI: −0.029, −0.243), accounting for 67.64% of the total effect. LPA identified three psychological capital profiles: low (40.71%), medium (44.84%), and high (14.45%). Total psychological capital significantly moderated the associations between work stressors and both WRQoL (*B* = 0.005, 95%CI: 0.002, 0.008) and innovative behavior (*B* = −0.006, 95%CI: −0.009, −0.004). In profile-specific analyses, the low-capital and high-capital profiles moderated the association between stressors and innovative behavior, whereas the medium profile did not show a significant moderating effect.

**Conclusion:**

Work stressors are negatively associated with innovative behavior among emergency and critical care nurses, and this association is partially accounted for by lower levels of WRQoL. Higher psychological capital, both as an overall resource and as distinct latent profiles, appears to attenuate the negative relationships between stressors and WRQoL as well as innovation. Managers might consider improving ED/ICU work environments, routinely monitoring WRQoL, and implementing stratified psychological capital interventions to support resilience and innovation.

## 1 Introduction

Innovation in nursing is a critical driver of service quality, patient safety and the sustainable development of healthcare systems. Nurses as the primary providers of bedside care are central to developing and implementing novel methods, technologies and care models that improve outcomes and advance the profession (Baig et al., 2022; Pennington and Driscoll, 2019). In the context of rapid technological change and mounting global health challenges, promoting sustained innovation among nurses is essential for optimizing care processes and meeting contemporary demands for high-quality care (Ma et al., 2023).

Emergency departments (EDs) and intensive care units (ICUs) are high-pressure clinical settings characterized by frequent high-stakes decisions, intense emotional labor and rapid technological change (Taskiran and Turk, 2023; Xie et al., 2024). Nurses working in these units commonly face prolonged standing, rotating shifts, heavy workloads and high turnover, producing greater cumulative stress than that experienced in many other wards (Liu S. et al., 2023; Wang M. et al., 2022; You et al., 2022). Evidence indicates that such stress adversely affects nurses’ physical and mental health and reduces work performance (Kong et al., 2024). Moreover, under high stress nurses may adopt conservative problem-solving and passive coping strategies that constrain idea generation and suppress innovative behavior (Wu et al., 2025; Zhou et al., 2024). Hence:

*Hypothesis 1*: Work stressors among emergency and critical care nurses were negatively associated with innovative behavior.

This prediction can be understood through two theoretical frameworks that, while distinct, lead to the same expectation. The Job Demands–Resources (JD–R) model describes a health-impairment pathway through which sustained demands gradually erode the resources that discretionary activities such as innovation require (Bakker and Demerouti, 2017). Conservation of Resources (COR) theory adds a complementary perspective: people are motivated above all to protect the resources they hold, and when those resources come under threat, they tend to narrow their efforts to what is essential (Hobfoll, 1989). Applied to the present context, the JD–R model points to resource erosion, while COR theory points to the behavioral withdrawal that follows. Both pathways converge on less innovative behavior under high stress.

Work-related quality of life (WRQoL) reflects the extent to which work satisfies individuals’ salient needs while enabling their contribution to organizational goals (Seid et al., 2025). The Job Demands–Resources (JD–R) model proposes that chronic job demands activate a health-impairment process that progressively drains employees of their physical and psychological resources (Bakker and Demerouti, 2017). Over time, this erosion becomes visible in indicators such as fatigue, reduced job satisfaction, and diminished well-being at work (Bakker and Demerouti, 2017; Kim and Yu, 2025). Within this framework, WRQoL can be understood as one such indicator—a composite signal reflecting how full or depleted a nurse’s resource reservoir has become. The demanding conditions of EDs and ICUs would be expected to drain this reservoir. Empirical studies report a robust negative association between work stress and nurses’ quality of working life (for example, *r* = −0.44, *p* < 0.001 in one hospital sample) (Al-Ruzzieh and Ayaad, 2021; Babapour et al., 2022; Diaz Hernandez et al., 2024). When that reservoir is depleted, COR theory suggests, nurses conserve what remains by directing their effort toward mandatory clinical tasks, while discretionary activities such as innovation are among the first to be set aside (Brunetto et al., 2022; Hobfoll et al., 2018). Accordingly:

*Hypothesis 2*: Work-related quality of life may mediate the association between work stressors and innovative behavior among emergency and critical care nurses.

Psychological capital — comprising self-efficacy, hope, resilience and optimism — is a key personal resource that facilitates coping under pressure (Hong and Zhonghua, 2010; Jia and Zhang, 2025). The JD–R model places such personal resources squarely in the role of a buffer: they help employees reappraise demanding situations and sustain goal-directed effort even when job pressures are high (Bakker and Demerouti, 2017). COR theory takes this a step further by specifying how the buffer works—individuals with larger reserves can draw on them to absorb losses elsewhere, interrupting the cycle of resource depletion before it spirals (Hobfoll, 1989). Research shows that higher psychological capital in nurses is associated with better performance, engagement and job satisfaction, and can buffer the negative impact of work stress (Kotb et al., 2024; Zhang et al., 2024). In innovation contexts, psychological capital has been linked to greater creativity and proactive behavior (Jiang and Zhou, 2020; Yan et al., 2020), yet most of this work has focused on direct associations, and whether psychological capital moderates the indirect pathway through work-related quality of life remains unclear. This buffering function is expected to operate at two points: first, by attenuating the negative link between work stressors and WRQoL; and second, by attenuating the direct link between work stressors and innovative behavior. Thus:

*Hypothesis 3*: Psychological capital may moderate the associations of work stressors with WRQoL and innovative behavior among emergency and critical care nurses.

Variable-centered analyses may mask meaningful heterogeneity in psychological capital. Latent Profile Analysis (LPA) can identify subgroups with distinct psychological capital configurations (e.g., low, medium, and high), which may differ in vulnerability to stress and in their propensity for innovation (Zhang et al., 2024). There is theoretical reason to take these subgroup differences seriously. COR theory predicts that people with scarce resources are highly sensitive to further loss and tend to react defensively, whereas those with ample resources are better able to absorb strain and remain proactive (Hobfoll et al., 2018; Russell et al., 2024). A variable-centered moderation test assumes that the buffering effect of psychological capital is roughly uniform across its full range—an assumption that may not hold if these qualitatively distinct response patterns are at work. Person-centered evidence can inform tailored, profile-specific interventions for workforce management. Therefore:

*Hypothesis 4*: Latent classes of psychological capital differentially moderate the relationships between work stressors and both WRQoL and innovative behavior.

**Figure 1:**
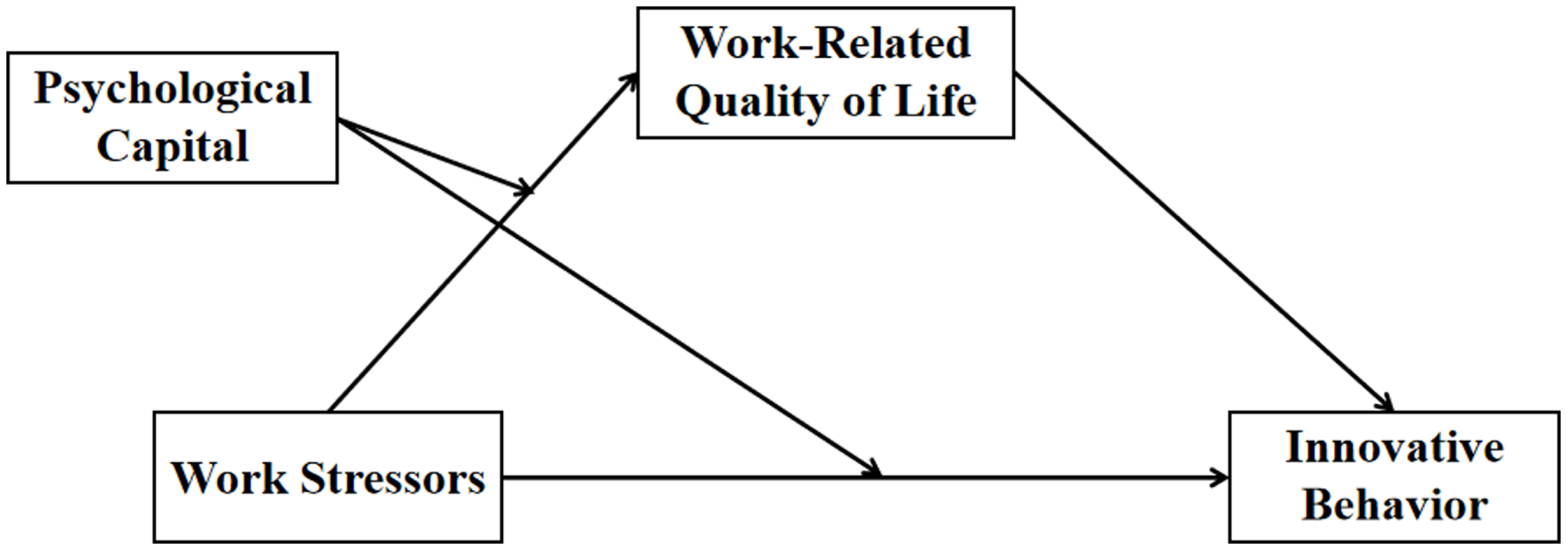
Moderated mediation model.

Grounded in JD–R and COR theoretical frameworks, this study tests a moderated-mediation model linking work stressors to innovative behavior via WRQoL, with psychological capital examined both as an overall moderator and as latent profiles. By integrating mediation/moderation analyses with LPA, the study aims to clarify mechanisms and heterogeneity that can guide targeted, practicable interventions to strengthen resilience and foster innovation among emergency and critical care nurses. The proposed model is shown in Figure 1.

## 2 Materials and methods

### 2.1 Setting and sample

This cross-sectional study was designed and reported following the STROBE guidelines for cross-sectional studies. In August 2024, a convenience sampling method was used to recruit emergency and critical care nurses from the emergency departments and intensive care units of three Grade-A tertiary general hospitals in Jinan and Zibo, Shandong Province. Inclusion criteria were: (1) possession of a valid People’s Republic of China Registered Nurse Qualification Certificate; (2) experience of independent shift work; and (3) informed consent to participate in this study. Exclusion criteria were: (1) nurses in advanced training; (2) student nurses; (3) nurses on study leave or vacation during the survey period; and (4) nurses who withdrew voluntarily or were unable to complete the study due to unforeseen events.

### 2.2 Measures

#### 2.2.1 Demographic and clinical characteristic

This self-designed instrument, developed by the researchers, collected the following demographic and professional characteristics: age, gender, marital status, educational level, years of experience, nursing seniority levels, professional position, type of employment contract, average monthly income, and night-shift work.

### 2.3 Chinese nurses stressor scale (CNSS)

The CNSS, adapted by Xiaomei and Yanjun (2000) from the original Gray-Toft and Anderson (1981) Nursing Stress Scale to reflect the Chinese context, comprises 35 items across five dimensions: Nursing Profession and Work (7 items), Workload and Time Allocation (5 items), Work Environment and Equipment (3 items), Patient Care (11 items), and Management and Interpersonal Relationships (9 items). Items are rated on a 4-point Likert scale from “strongly disagree” (1) to “strongly agree” (4), yielding a total score range of 35–140, with higher scores indicating greater perceived work stress. In the present study, the overall Cronbach’s *α* for the CNSS was 0.963.

### 2.4 Work-related quality of life scale (WRQOL)

The WRQOL, adapted by Ya et al. (2014) to suit the Chinese context, comprises 33 items across seven dimensions: Job Evaluation (5 items), Career Satisfaction (5 items), Control at Work (5 items), Work–Family Balance (2 items), Working Conditions (6 items), General Well-Being (5 items), and Work-Related Stress (5 items). Items are rated on a 5-point Likert scale from “strongly disagree” (1) to “strongly agree” (5), yielding a total score range of 33–165, with higher scores indicating better work-related quality of life. In this study, the overall Cronbach’s *α* for the WRQOL was 0.917.

### 2.5 Psychological capital questionnaire (PCQ)

The Chinese version of the Nurse Psychological Capital Questionnaire (PCQ), adapted by Hong and Zhonghua (2010) to fit the Chinese context, comprises four dimensions: self-efficacy (6 items), hope (6 items), resilience (5 items), and optimism (3 items). Items are rated on a 6-point Likert scale ranging from “strongly disagree” (1) to “strongly agree” (6), yielding a total score range of 20–120, with higher scores indicating greater levels of psychological capital. In this study, the overall Cronbach’s *α* for the PCQ was 0.973.

### 2.6 Innovative behavior inventory (IBI)

The Chinese version of the Innovative Behavior Inventory (IBI), adapted by Liming et al. (2021) for the Chinese context, comprises five dimensions: Idea Generation and Exploration (6 items), Planning, Communication, and Implementation (5 items), Acquisition of Human Resources (3 items), Overcoming Obstacles (3 items), and Clinical Application (3 items), for a total of 20 items. Items are rated on a 5-point Likert scale from “strongly disagree” (1) to “strongly agree” (5), yielding a total score range of 20–100, with higher scores indicating greater levels of innovative behavior. In this study, the overall Cronbach’s α for the IBI was 0.972.

### 2.7 Data collection

Prior to data collection, the investigators first liaised with the Nursing Departments of the participating hospitals to present the study design and obtain permission from the nursing administrators. Eligible participants were identified in accordance with the inclusion and exclusion criteria. After explaining the study’s purpose and securing informed consent, participants were provided with either a paper-based or electronic questionnaire. All questionnaires were accompanied by standardized instructions, and every item was designated as mandatory; participants were informed that any questions could be addressed in person, by telephone, or via WeChat. Upon receipt of the completed questionnaires, the researchers immediately reviewed paper forms for missing responses, patterned answering, or other irregularities, and screened electronic submissions for similar response patterns as well as excessively short completion times (<5 min). Any questionnaires exhibiting these issues were excluded from the analysis.

### 2.8 Data analysis

The required sample size was determined *a priori*. According to the Monte Carlo simulation results of Fritz and Mackinnon (2007), detecting a medium-sized mediation effect (*a* = 0.39, *b* = 0.39) with 0.80 statistical power at *α* = 0.05 requires approximately 462 participants. In addition, simulation evidence (Nylund et al., 2007) indicates that samples exceeding 500 are adequate for latent profile analysis. Anticipating approximately 10% invalid or incomplete responses, we aimed to recruit at least 515 nurses. Data were entered and organized in Excel, and statistical analyses were performed using SPSS 26.0 and Mplus 8.3. Categorical variables were summarized as counts and percentages (%), and continuous variables as means ± standard deviations. Pearson correlation analysis was used to assess relationships among variables. Mediation effects were tested using the PROCESS macro (version 4.1) for SPSS, specifying Model 4. Latent profile analysis (LPA) was conducted in Mplus 8.3 using the 20 items of the Psychological Capital Questionnaire as manifest indicators. Models with one- to four-profiles were sequentially fitted, and model parameters were compared using the following fit indices: (1) information criteria—Akaike’s information criterion (AIC), Bayesian information criterion (BIC), and sample-size-adjusted BIC (aBIC)—with lower values indicating better fit; (2) classification accuracy—entropy values closer to 1 denote more precise classification; and (3) likelihood ratio tests—the Lo–Mendell–Rubin adjusted LRT (LMR) and the bootstrap likelihood ratio test (BLRT), where *p* < 0.05 indicates that a K-profile model fits significantly better than a K–1-profile model. Additionally, each latent class in the optimal model was required to comprise at least 5% of the total sample. The hypothesized moderated mediation model was tested using the PROCESS macro for SPSS (Model 8). All continuous predictor variables were mean-centered prior to the analysis to reduce multicollinearity and facilitate the interpretation of interaction effects. Significant interactions were probed by estimating simple slopes at one standard deviation above and below the mean of the moderator. Bootstrap confidence intervals (95% CI) based on 5,000 resamples were used to evaluate the significance of indirect and conditional effects. Finally, moderation analyses were conducted separately within each psychological capital profile using PROCESS Model 8. Statistical significance was set at *α* = 0.05.

### 2.9 Ethical considerations

This study was approved by the Ethics Committee of Qilu Hospital, Shandong University (Approval No. KYLL-202405-001-1). The Declaration of Helsinki was followed throughout the research. All participants were informed about the study procedures and gave informed consent. They were assured of confidentiality and of their right to withdraw at any time.

## 3 Results

### 3.1 Common method bias test

A Harman single-factor test was conducted with all items from the four scales entered into an unrotated exploratory factor analysis. The first factor accounted for 35.15% of the total variance, below the 50% threshold (Podsakoff et al., 2003).

### 3.2 Sample characteristics

A total of 579 nurses were invited to participate, of whom 548 returned the questionnaire. After excluding 15 questionnaires (8 due to patterned responses, 4 due to completion times under 5 min, and 3 due to excessive missing data), 533 nurses were included in the final analysis (effective response rate: 92.1%). The mean age was 30.36 ± 6.19 years, and the mean years of nursing experience was 7.79 ± 6.61. The sample comprised 384 female nurses (72.0%) and 298 married nurses (55.9%). A total of 496 nurses (93.1%) held a bachelor’s degree. Regarding nursing levels, 147 nurses (27.6%) were classified as N2 and 173 (32.5%) as N3; 257 nurses (48.2%) held the professional title of Nurse Practitioner. Clinically, 447 nurses (83.9%) served as bedside clinical nurses. In terms of employment, 479 nurses (89.9%) were on fixed-term contracts. Monthly income exceeded ¥10,000 for 220 nurses (41.3%), and 444 nurses (83.3%) routinely worked night shifts. Detailed demographic and professional characteristics are presented in Table 1.

**Table 1:** Participant characteristics (*n* = 533).

Characteristics	*M* ± SD/*N*(%)
Age	30.36 ± 6.19
Gender	
Male	149(28.0%)
Female	384(72.0%)
Years of experience	7.79 ± 6.61
Marital status	
Married	298(55.9%)
Others	235(44.1%)
Education level	
Junior college or less	17(3.2%)
Bachelor	496(93.1%)
Master or above	20(3.7%)
Nursing seniority levels	
N1	111(20.8%)
N2	147(27.6%)
N3	173(32.5%)
N4	102(19.1)
Professional title	
Registered Nurse	123(23.1%)
Nurse Clinician	257(48.2%)
Senior Nurse	147(27.6%)
Associate/Chief Nurse	6(1.1%)
Position	
Nurse	447(83.9%)
Charge Nurse	45(8.4%)
Clinical Preceptor	32(6.0%)
Head Nurse	9(1.7%)
Employment type	
Permanent staff	27(5.1%)
Human agency	13(2.4%)
Contractor	479(89.9%)
Labor dispatch	14(2.6%)
Average monthly income	
<4,000	29(5.4%)
4,000 ~ 8,000	139(26.1%)
8,000 ~ 10,000	145(27.2%)
>10,000	220(41.3%)
Having night shifts	
Yes	444(83.3%)
No	89(16.7%)

### 3.3 Correlation analysis

The mean score for work stressors among emergency and critical care nurses was 84.51 ± 17.74; for work-related quality of life, 121.47 ± 15.14; for psychological capital, 88.02 ± 16.59; and for innovative behavior, 67.95 ± 14.01. The work stressor score among emergency and critical care nurses was negatively correlated with work-related quality of life, psychological capital, and innovative behavior (all *p* < 0.01). Work-related quality of life was positively correlated with psychological capital and innovative behavior (both *p* < 0.01), and psychological capital was positively correlated with innovative behavior (*p* < 0.01). Detailed coefficients are provided in Table 2.

**Table 2:** Descriptive statistics and correlations among study variables (*n* = 533).

Variables	Work stressors	Work-related quality of life	Psychological capital	Innovative behavior
Work Stressors (84.51 ± 17.74)	1			
Work-Related Quality of Life (121.47 ± 15.14)	−0.502	1		
Psychological Capital (88.02 ± 16.59)	−0.443	0.632	1	
Innovative Behavior (67.95 ± 14.01)	−0.356	0.558	0.579	1

All *p*-values < 0.01.

### 3.4 Mediating effect analysis

The mediation effect of work-related quality of life in the relationship between work stressors and innovative behavior was tested using SPSS PROCESS Model 4. Results indicated that work stressors were significantly associated with lower work-related quality of life (*β* = −0.419, *t* = −12.739, *p* < 0.001), and higher work-related quality of life was significantly associated with higher innovative behavior (*β* = 0.445, *t* = 11.801, *p* < 0.001). In addition, work stressors were significantly associated with lower innovative behavior after accounting for work-related quality of life (*β* = −0.089, *t* = −2.738, *p* = 0.006). Work-related quality of life partially mediated the effect of work stressors on innovative behavior, with an indirect effect of −0.419 × 0.445 = −0.186 (95% CI: −0.243 to −0.131). The ratio of the indirect effect to the total effect was 67.64%. Mediation analysis results are presented in Table 3.

**Table 3:** Mediating effect of work-related quality of life between work stressors and innovative behavior (*n* = 533).

Effect Type	Mediating pathway	Effect size	LLCI	ULCI	Effect proportion (%)
Direct Effect	Work Stressors → Innovative Behavior	−0.089	0.032	−0.152 ~ −0.025	32.36
Indirect Effect	Work Stressors → Work-Related Quality of Life →Innovative Behavior	−0.186	0.029	−0.243 ~ −0.131	67.64
Total Effect	Work Stressors → Innovative Behavior	−0.275	0.032	−0.338 ~ −0.213	100

Control variables included age, gender, marital status, education level, years of experience, nursing, seniority levels, professional title, position, employment type, average monthly income, having night shifts.

### 3.5 Latent profile analysis

Latent profile analysis was performed using the 20 item scores of the Psychological Capital Questionnaire; the model fit indices are presented in Table 4. As the number of profiles increased from one to four, the values of the Akaike information criterion (AIC), Bayesian information criterion (BIC), and sample-size-adjusted BIC (aBIC) progressively decreased, indicating improved model fit. The three-profile solution achieved the highest entropy value, reflecting optimal classification accuracy, whereas the four-profile solution yielded a non-significant Lo–Mendell–Rubin adjusted likelihood ratio test (LMR; *p* > 0.05). In contrast, both the LMR and the bootstrap likelihood ratio test (BLRT) were statistically significant for the three-profile model (*p* < 0.05), demonstrating superior fit relative to the two-profile model. Moreover, each latent class in the three-profile solution comprised at least 5% of the total sample. On the basis of these criteria, emergency and critical care nurses’ psychological capital was best represented by three distinct latent profiles.

**Table 4:** Model fit indices for latent classes of nurses’ psychological capital (*n* = 533).

Latent class	AIC	BIC	aBIC	Entropy	LMRT (P)	BLRT (P)	Latent class probability (%)
1	30617.010	30788.151	30661.178				
2	25292.553	25553.543	25359.910	0.956	0.001	<0.001	52.92/47.08
3	23381.846	23732.685	23472.391	0.966	0.003	<0.001	40.71/44.84/14.45
4	22176.437	22617.125	22290.171	0.957	0.732	<0.001	12.57/35.84/37.71/13.88

**Figure 2:**
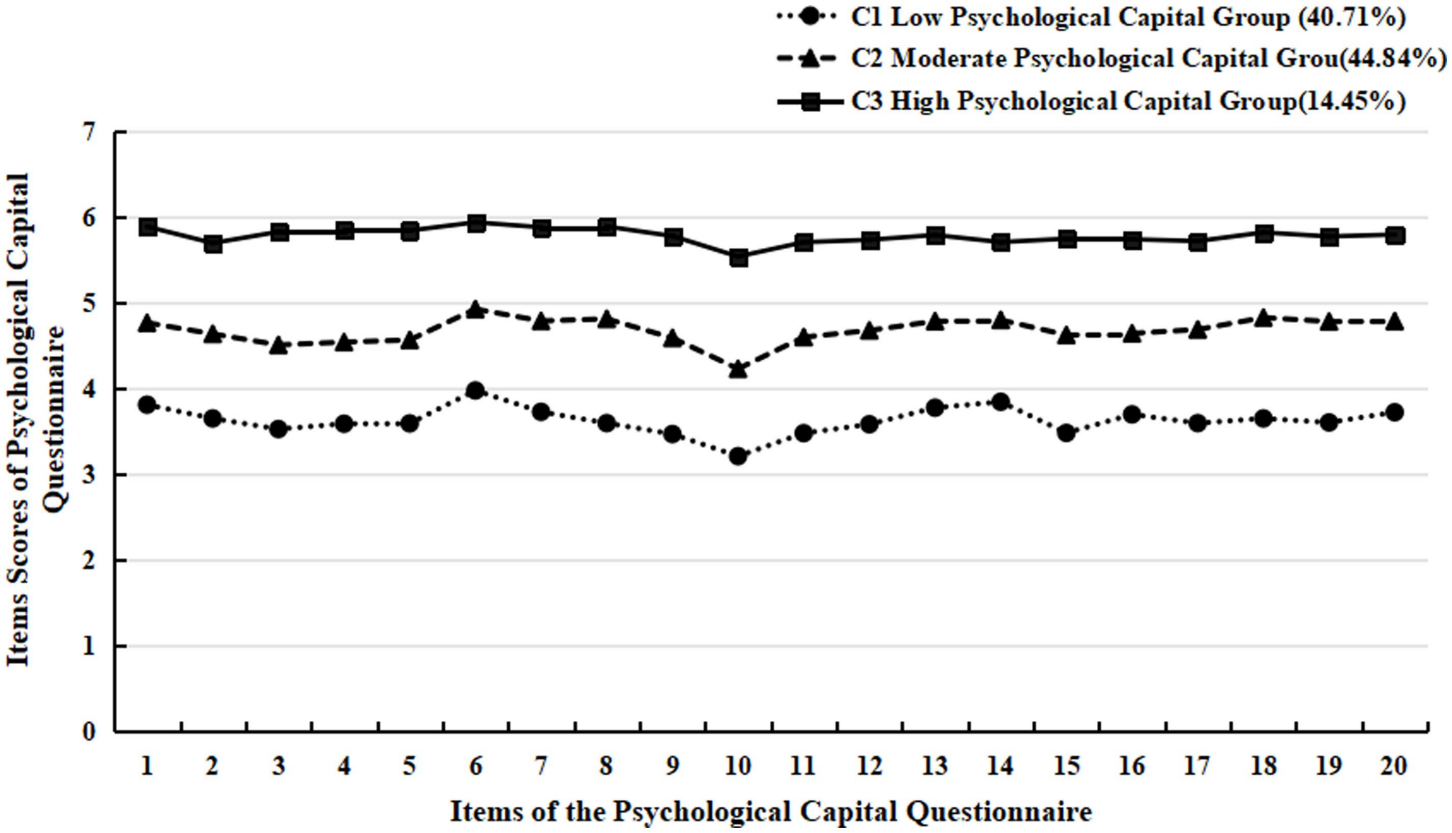
Latent profile characteristics of psychological capital in emergency and critical care nurses.

A profile plot of the three latent classes of psychological capital was generated based on their mean item scores (Figure 2). All three profiles exhibited similar trajectory shapes with no intersections. The classes were labeled as follows: Class C1 (*n* = 217): Mean item score = 3.62 ± 0.45, representing the lowest level of psychological capital; labeled the Low Psychological Capital group. Class C2 (*n* = 239): Mean item score = 4.67 ± 0.30, representing a moderate level of psychological capital; labeled the Moderate Psychological Capital group. Class C3 (*n* = 77): Mean item score = 5.78 ± 0.25, representing the highest level of psychological capital; labeled the High Psychological Capital group.

### 3.6 Moderating effect analysis

**Figure 3:**
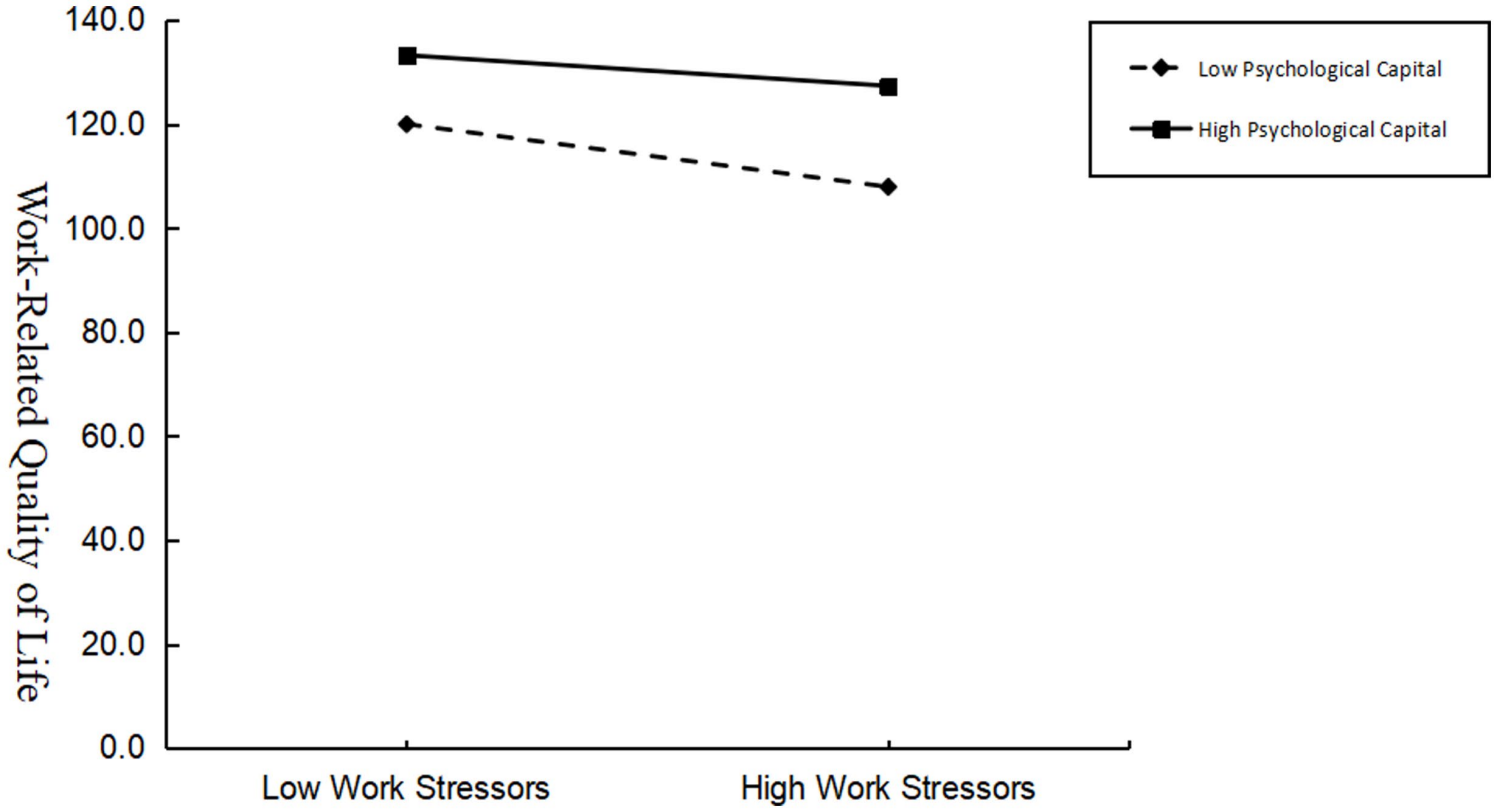
The moderating effect of psychological capital on the relationship between work stressors and work-related quality of life.

**Figure 4:**
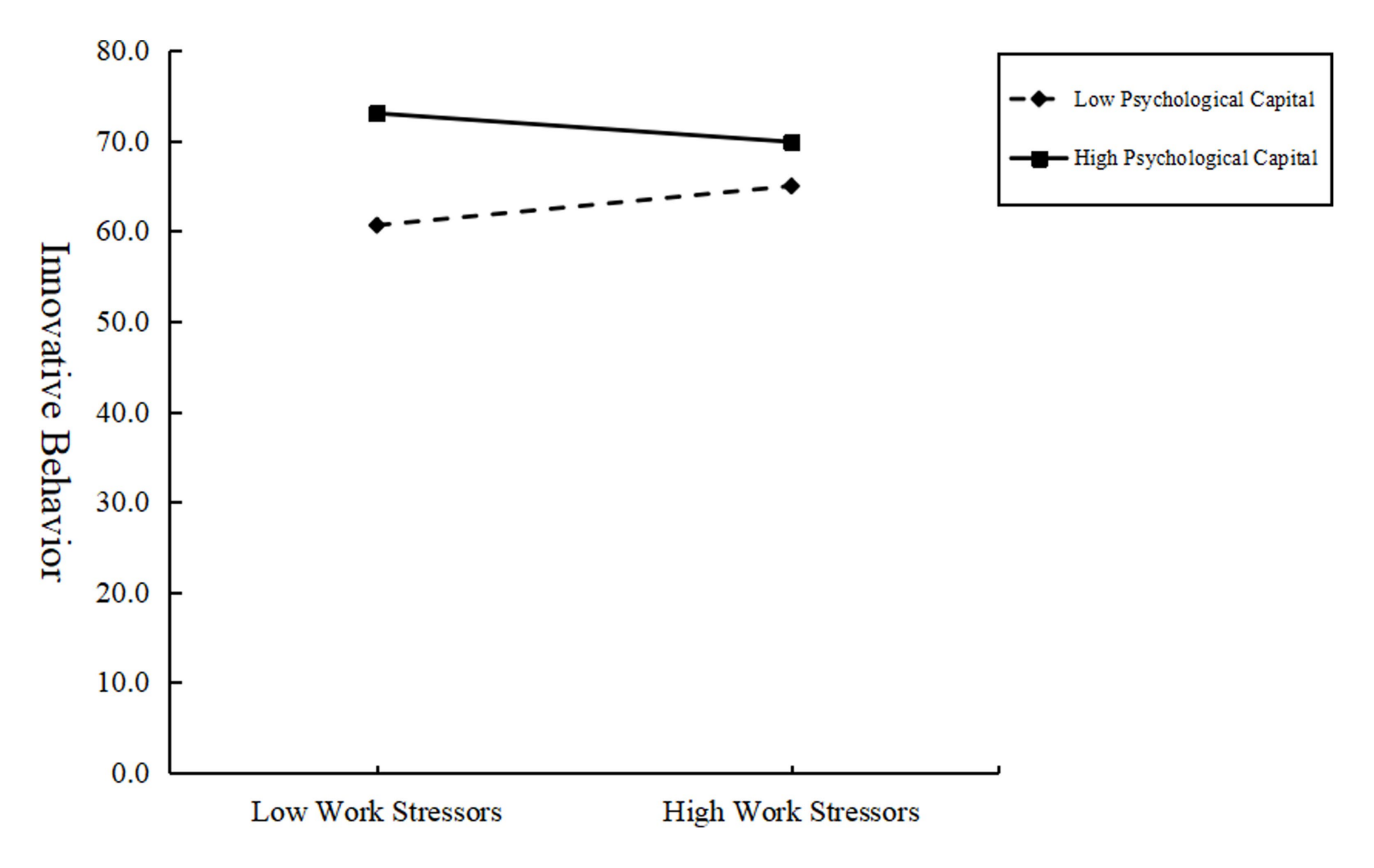
The moderating role of psychological capital in the relationship between work stressors and innovative behavior.

The interaction between work stressors and psychological capital significantly was significantly associated with work-related quality of life (*B* = 0.005, *t* = 3.618, *p* < 0.01) and innovative behavior (*B* = −0.006, *t* = −4.539, *p* < 0.01). Simple slope analyses were conducted at one standard deviation above and below the mean of psychological capital. For the path from work stressors to work-related quality of life, the negative association was significant both at low psychological capital (Effect = −0.335, *t* = −7.253, *p* < 0.01) and at high psychological capital (Effect = −0.175, *t* = −5.397, *p* < 0.01). For the path from work stressors to innovative behavior, the simple slope was positive at low psychological capital (Effect = 0.119, *t* = 2.531, *p* < 0.05) and negative at high psychological capital (Effect = −0.078, *t* = −2.413, *p* < 0.05) (see Table 5 and Figures 3, 4).

**Table 5:** Moderating effect of psychological capital (*n* = 533).

Variables	Model 1		Model 2	
	Outcome variable: work-related quality of life		Outcome variable: innovative behavior	
	*B*(95%CI)	*t*	*B*(95%CI)	*t*
Work Stressors	−0.709(−0.986, −0.432)	−5.026^**^	0.578(0.304, 0.853)	4.136^**^
Psychological Capital	0.053(−0.196, 0.302)	0.419	0.800(0.559, 1.040)	6.527^**^
Work-Related Quality of Life			0.318(0.234, 0.401)	7.471^**^
Work Stressors × Psychological Capital	0.005(0.002, 0.008)	3.618^**^	−0.006(−0.009, −0.004)	−4.539^**^
Age	−0.330(−0.851, 0.191)	−1.246	0.054(−0.451, 0.558)	0.208
Gender	0.111(−2.067, 2.290)	0.100	−1.518(−3.627, 0.591)	−1.414
Marital Status	−1.548(−4.367, 1.272)	−1.078	1.577(−1.154, 4.309)	1.134
Education level	1.360(−2.558, 5.277)	0.682	−2.505(−6.298, 1.289)	−1.297
Years of experience	0.256(−0.227, 0.740)	1.041	0.221(−0.247, 0.690)	0.927
Nursing seniority levels	0.885(−1.044, 2.813)	0.901	−1.422(−3.290, 0.446)	−1.496
Professional title	−1.436(−3.764, 0.891)	−1.212	−0.729(−2.985, 1.527)	−0.635
Position	0.679(−1.051, 2.409)	0.771	1.647(−0.028, 3.323)	1.931
Employment type	2.033(−0.213, 4.280)	1.778	−0.274(−2.455, 1.907)	−0.247
Average monthly income	−0.384(−1.769, 1.002)	−0.544	−0.804(−2.145, 0.536)	−1.179
Having night shifts	2.925(−0.008, 5.858)	1.959	−1.520(−4.369, 1.329)	−1.048
*R^2^*	0.489		0.443	
*F*	35.406^**^		27.361^**^	

Control variables included age, gender, marital status, education level, years of experience, nursing seniority levels, professional title, position, employment type, average monthly income, and having night shifts. ^**^p < 0.01.

Moderation analyses were conducted separately for the three psychological capital profiles using SPSS PROCESS Model 8 (Table 6). In the Low profile, the interaction was significantly associated with both work-related quality of life (*B* = −0.219, *t* = −3.081, *p* < 0.01) and innovative behavior (*B* = 0.296, *t* = 4.633, *p* < 0.01). In the Moderate profile, the interaction term was significantly associated with work-related quality of life (*B* = 0.282, *t* = 4.050, *p* < 0.01) but not innovative behavior. In the High profile, the interaction significantly term was significantly associated with both work-related quality of life (*B* = 0.347, *t* = 4.743, *p* < 0.01) and innovative behavior (*B* = −0.173, *t* = −2.555, *p* < 0.05).

**Table 6:** Moderating effect in the low psychological capital group (*n* = 533).

Variables	Low psychological capital group				Moderate psychological capital group				High psychological capital group			
	Model 3		Model 4		Model 5		Model 6		Model 7		Model 8	
	Outcome variable: Work-related quality of life		Outcome variable: Innovative Behavior		Outcome variable: Work-related quality of life		Outcome variable: Innovative Behavior		Outcome variable: Work-related quality of life		Outcome variable: Innovative Behavior	
	*B*(95%CI)	*t*	*B*(95%CI)	*t*	*B*(95%CI)	*t*	*B*(95%CI)	*t*	*B*(95%CI)	*t*	*B*(95%CI)	*t*
Work Stressors	−0.263(−0.338, −0.188)	−6.879^**^	−0.144(−0.213, −0.074)	−4.038^**^	−0.498(−0.573, −0.423)	−13.042^**^	−0.115(−0.191, −0.039)	−2.978^*^	−0.403(−0.481, −0.325)	−10.192^**^	0.007(−0.07, 0.084)	0.189
Psychological Capital	9.219(−3.294, 21.732)	1.447	−30.88(−42.065, −19.695)	−5.424^**^	−20.54(−32.256, −8.825)	−3.444^**^	−5.403(−15.807, 5.001)	−1.02	−10.996(−21.902, −0.089)	−1.981^*^	0.402(0.324, 0.48)	10.131^**^
Work-Related Quality of Life			0.408(0.331, 0.485)	10.405^**^			0.433(0.357, 0.508)	11.213^**^			20.347(10.452, 30.243)	4.039^**^
Work Stressors × Psychological Capital	−0.219(−0.358, −0.079)	−3.081^**^	0.296(0.171, 0.422)	4.633^**^	0.282(0.145, 0.418)	4.050^**^	0.076(−0.046, 0.197)	1.219	0.347(0.203, 0.491)	4.743^**^	−0.173(−0.305, −0.04)	−2.555^*^
Age	−0.16(−0.738, 0.417)	−0.546	0.206(−0.31, 0.721)	0.784	−0.182(−0.792, 0.427)	−0.588	0.231(−0.304, 0.767)	0.848	−0.292(−0.869, 0.285)	−0.994	0.11(−0.412, 0.632)	0.413
Gender	−0.689(−3.106, 1.729)	−0.56	−1.853(−4.01, 0.304)	−1.687	−1.008(−3.554, 1.539)	−0.777	−1.948(−4.186, 0.289)	−1.711	−0.675(−3.084, 1.735)	−0.55	−1.753(−3.932, 0.426)	−1.58
Marital Status	0.214(−2.913, 3.34)	0.134	2.049(−0.74, 4.838)	1.443	1.241(−2.037, 4.519)	0.744	3.104(0.224, 5.983)	2.117^*^	−0.355(−3.465, 2.755)	−0.224	2.625(−0.186, 5.437)	1.835
Education level	−0.984(−5.341, 3.372)	−0.444	−4.015(−7.902, −0.128)	−2.029^*^	−1.289(−5.884, 3.306)	−0.551	−3.265(−7.301, 0.771)	−1.589	1.831(−2.535, 6.197)	0.824	−2.805(−6.754, 1.144)	−1.395
Years of experience	0.211(−0.326, 0.749)	0.773	0.122(−0.357, 0.602)	0.501	0.26(−0.307, 0.827)	0.901	0.143(−0.355, 0.641)	0.563	0.303(−0.233, 0.839)	1.112	0.204(−0.281, 0.689)	0.825
Nursing seniority levels	0.08(−2.060, 2.220)	0.073	−1.86(−3.769, 0.049)	−1.914	−0.597(−2.845, 1.65)	−0.522	−1.908(−3.883, 0.066)	−1.899	0.222(−1.913, 2.357)	0.204	−1.645(−3.575, 0.284)	−1.675
Professional title	1.082(−0.842, 3.005)	1.105	1.677(−0.041, 3.395)	1.917	1.261(−0.763, 3.284)	1.224	2.021(0.242, 3.8)	2.232^*^	0.861(−1.054, 2.776)	0.883	1.805(0.072, 3.537)	2.046^*^
Position	−1.173(−3.768, 1.421)	−0.888	−0.005(−2.322, 2.311)	−0.005	−0.915(−3.655, 1.825)	−0.656	−0.345(−2.752, 2.062)	−0.282	−0.806(−3.383, 1.772)	−0.614	−0.516(−2.846, 1.815)	−0.435
Employment type	1.427(−1.065, 3.919)	1.125	−0.404(−2.63, 1.822)	−0.356	0.154(−2.457, 2.765)	0.116	−0.576(−2.869, 1.716)	−0.494	1.968(−0.538, 4.474)	1.543	−0.516(−2.787, 1.755)	−0.447
Average monthly income	−1.357(−2.894, 0.18)	−1.735	−1.12(−2.495, 0.255)	−1.6	−1.292(−2.901, 0.318)	−1.577	−1.604(−3.021, −0.188)	−2.225^*^	−0.545(−2.076, 0.985)	−0.7	−1.057(−2.441, 0.327)	−1.5
Having night shifts	2.405(−0.854, 5.664)	1.45	−2.154(−5.067, 0.759)	−1.453	1.787(−1.643, 5.217)	1.023	−2.515(−5.53, 0.499)	−1.639	2.911(−0.344, 6.166)	1.757	−1.977(−4.928, 0.974)	−1.316
*R^2^*	0.368		0.415		0.299		0.370		0.373		0.403	
*F*	21.573^**^		24.405^**^		15.747^**^		20.251^**^		21.983^**^		23.274^**^	

Control variables included age, gender, marital status, education level, years of experience, nursing, seniority levels, professional title, position, employment type, average monthly income, having night shifts. *p < 0.05, **p < 0.01.

## 4 Discussion

The results of this study show that emergency and critical care nurses reported a moderate level of innovative behavior, and work stressors were significantly and negatively associated with innovative behavior. Mediation analyses indicated that work-related quality of life partially mediated the stress-to-innovation relationship. Variable-centered moderation analyses demonstrated that overall psychological capital buffered the adverse effects of work stressors on both work-related quality of life and innovative behavior. Complementary person-centered latent profile analysis identified three psychological-capital profiles (low, medium, and high), with profile membership differentially moderating the stress–outcome links: the low- and high-capital groups showed significant moderating effects, whereas the medium group did not, highlighting that both the level and configuration of personal resources influence nurses’ vulnerability or resilience to work stress in relation to innovation.

### 4.1 The current status of innovative behavior among emergency and critical care nurses

In the present study, the mean total score for innovative behavior among emergency and critical care nurses was 67.95 ± 14.01, indicating a moderate level. This moderate level of innovation is consistent with findings from ICU nurses in other parts of China (Ge et al., 2025) and from nurses in several other countries, including Greece (Moisoglou et al., 2024), Turkey (Agaoglu et al., 2025), and Egypt (Awad et al., 2025). Some studies, however, point to higher scores—for instance, among oncology nurses in Jinan (Jing, 2024) and among emergency nurses in Alexandria (Awad et al., 2025). The gap between these findings and the moderate levels observed in our and other ICU samples likely reflects the particularly severe resource constraints and task urgency that characterize ED and ICU work (Han et al., 2024). In the Chinese context, these pressures are further compounded by high patient volumes, staffing shortages, and frequent night shifts. Differences in nursing education systems and continuing professional development models across countries may also contribute.

### 4.2 The relationship between work stressors and innovative behavior among emergency and critical care nurses

The present findings revealed a significant negative correlation between work stressors and innovative behavior among emergency and critical care nurses (*p* < 0.01), supporting Hypothesis H1. This result is consistent with the broader literature, which has identified work stress as a major barrier to innovative behavior in this population (Anjum and Zhao, 2022; Junjie et al., 2023; Yang and Wu, 2021). In the uniquely demanding environments of emergency departments and intensive care units, nurses face a wide range of stressors—excessive workloads, rapidly fluctuating patient conditions, and difficult interactions with patients and families, among others (Vizeshfar et al., 2022). These persistent demands not only deplete nurses’ physical and emotional resources, but may also reinforce habitual thinking patterns, leaving little time or energy for creative problem solving and experimentation. Heightened stress can further strengthen reliance on established routines and foster a fear of change, thereby suppressing innovative behavior (Bao et al., 2024). At the same time, some evidence suggests that the effects of stress are not uniform: when certain work demands are appraised as challenges rather than threats, they may to some extent stimulate innovative effort (Kim and Lee, 2024; Leung et al., 2011). This highlights the importance of nuanced stress management strategies that help nurses distinguish between different types of stressors, rather than treating all stress as inherently detrimental.

### 4.3 The mediating role of work-related quality of life

This study found that work-related quality of life mediated the relationship between work stressors and innovative behavior, supporting Hypothesis H2. In the high-pressure environments of EDs and ICUs, sustained work stress erodes nurses’ work-related quality of life, often manifesting as reduced job satisfaction and difficulty balancing work and family life (Skoufi et al., 2017), which in turn diminishes both their motivation and capacity for innovation. A systematic review of emergency department nurses has confirmed that stress and burnout are closely linked to poorer professional quality of life (Jachmann et al., 2025), and Mohammadi et al. (2022) similarly reported a significant negative correlation between job stress and quality of work-life among nurses. The present study further reveals that the decline in work-related quality of life extends beyond personal well-being to affect innovative behavior. A survey of nurses in Greece showed that those who perceived stronger organizational support for innovation also reported higher levels of innovative behavior (Moisoglou et al., 2024), lending additional weight to the association between work-related quality of life and nurses’ innovative efforts. These findings are consistent with those of Jianfeng et al. (2013), who identified work stress as a critical determinant of health workers’ quality of life and noted that improvements in well-being often stimulate creative thinking.

### 4.4 The moderating role of psychological capital

The findings suggest that psychological capital buffers the detrimental effects of work stressors on both work-related quality of life (WRQoL) and innovative behavior at the overall level, while also exhibiting significant heterogeneity at the individual level, supporting hypotheses H3 and H4. In general, under comparable levels of work stress, nurses with low psychological capital experience the greatest decline in work-related quality of life (WRQoL), whereas those with high Psychological capital are better able to maintain stability. The buffering role of psychological capital has been observed across different healthcare systems: among Australian nurses, psychological capital was found to moderate the negative impact of job demands and role stress on work engagement (Grover et al., 2018). This finding suggests that abundant psychological resources can mitigate the negative consequences of resource depletion triggered by stressors (Liu Y. et al., 2023; Rashnoudi et al., 2025; Wang M. F. et al., 2022).

The person-centered latent profile analysis (LPA) further revealed distinct moderating patterns across the three psychological capital profiles (low, moderate, and high). All profiles showed significant moderation in the stress–WRQoL pathway, but their effects on the stress–innovation link diverged. In the low psychological capital group, a positive simple slope was observed between work stressors and innovative behavior—a pattern that appears inconsistent with the overall negative association found in the full sample. One possible theoretical account, consistent with COR theory, is that individuals with very limited resources may at times respond to stressors by investing effort into extra-role activities such as innovation, in an attempt to generate new resources that could offset their current deficit (Hobfoll, 1989; Hobfoll et al., 2018). This interpretation aligns with the challenge stressor hypothesis, which posits that certain stressors can be appraised as opportunities for growth and thereby elicit proactive behavior (Cai et al., 2022; Dou et al., 2022; Wei et al., 2024). However, given the cross-sectional design, caution is warranted: the positive slope could also reflect reverse causation, whereby nurses who engage in more innovative activities encounter greater stress as a consequence, or the influence of unmeasured third variables.

In contrast, nurses in the high psychological capital group showed a negative stress–innovation relationship. This finding fits more squarely within the standard prediction of COR theory—namely, that individuals prioritize protecting existing resources over acquiring new ones when under threat (Hobfoll, 1989). For nurses with abundant psychological reserves, sustained stress may signal that it is prudent to channel effort into preserving core clinical functioning rather than expending additional resources on discretionary innovation (Cai et al., 2022; Liu et al., 2025). This pattern is consistent with evidence from Iraqi nurses, among whom psychological capital positively predicted innovative work behavior (Alwali, 2024), suggesting that when psychological resources are preserved, nurses are more likely to engage in innovation. From this perspective, one possible interpretation is that both the low- and high-capital groups may reflect resource-driven adaptation processes: in both cases, behavior is oriented toward eventual resource balance. The difference lies in the starting level of resources, which shapes whether the optimal strategy is to invest further or to conserve.

The moderate-psychological capital group showed no significant moderating effect, a null finding that may reflect a resource level insufficient to trigger strong compensatory behavior yet not abundant enough to elicit a clear resource-conservation response. Together, these three patterns suggest that the stress–innovation relationship is not fixed but varies systematically as a function of the resources a nurse holds—a proposition central to COR theory yet rarely tested with person-centered methods in this population. Longitudinal studies are needed to clarify the temporal dynamics underlying these different patterns.

### 4.5 Implications for practice

Several practical implications follow from these findings, spanning clinical practice, nursing management, and education. In clinical practice, work-related quality of life can serve as an early warning signal. Declining job satisfaction or mounting work–family conflict may indicate that a nurse’s capacity for innovation is already under strain. Routine monitoring of WRQoL with brief, validated tools could help identify nurses at risk before their innovative behavior declines. Nurses with low psychological capital, who in our data showed a positive stress–innovation slope, warrant particular attention: their initial burst of innovation effort under stress may not be sustainable and could give way to exhaustion if stressors are not addressed.

For nursing management and workforce policy, the three psychological capital profiles point toward differentiated strategies rather than uniform approaches. Low-capital nurses may gain most from resilience training, self-efficacy building, and one-to-one mentoring. Moderate-capital nurses could benefit from team-based support and scenario-based simulations. High-capital nurses, who tended to withdraw from innovation under sustained stress, may need flexible scheduling, research opportunities, or mentoring roles that preserve their core resources while sustaining engagement. More broadly, these findings reinforce calls for staffing models that account for the psychological demands of ED and ICU work, alongside conventional nurse-to-patient ratios.

In education and training, the malleability of psychological capital offers a promising lever. Brief educational programs have been shown to improve both psychological capital and innovative behavior in nurses (Kotb et al., 2024). Embedding psychological capital training into orientation programs for new ED and ICU nurses, and offering refresher workshops that help nurses distinguish challenge stressors from hindrance stressors, could contribute to building a workforce that is both resilient and innovative. Scenario-based simulations that expose nurses to realistic high-stress situations while equipping them with psychological resources may also prove valuable.

### 4.6 Limitations

Several limitations should be noted. First, all measures were self-reported and collected at a single time point. Both paper-based and electronic questionnaires were used. For the electronic version, although each IP address was restricted to a single submission, we could not independently verify whether respondents met the inclusion criteria, nor could we entirely rule out the possibility that a participant completed the survey on different devices. Common method variance may have inflated some of the observed associations. The cross-sectional design also prevents causal inference; the positive stress–innovation slope observed in the low psychological capital group, for instance, could reflect reversed causation. Multi-wave designs incorporating objective indicators of innovation would help address these concerns. Second, the sample was relatively homogeneous: over 93% of participants held a bachelor’s degree, nearly 84% regularly worked night shifts, and approximately 90% were on contract-based employment. While this profile is representative of emergency and critical care nurses in Chinese tertiary hospitals, generalisability to nurses with different educational backgrounds, employment arrangements, or those in primary and secondary care settings may be limited. The study also drew participants from three hospitals in Shandong Province, and the reliance on convenience sampling may have introduced selection bias—nurses who were available and willing to participate during the survey period may differ from those who were not. Finally, this study relied solely on quantitative measures. Qualitative research exploring how emergency and critical care nurses experience work stress and innovation in their daily practice could add depth to the understanding of these phenomena. All measures were validated in Chinese populations; cross-cultural measurement invariance remains untested, and replication in other cultural contexts is warranted.

## 5 Conclusion

This study examined how work stressors, work-related quality of life, and psychological capital relate to innovative behavior among emergency and critical care nurses. The findings indicated that innovative behavior was at a moderate level, that work stressors were negatively associated with innovative behavior, and that work-related quality of life partially explained this association. Psychological capital—both as an overall resource and as distinct latent profiles—appeared to attenuate the negative links between work stressors and the two outcomes. Grounded in the JD–R model and COR theory, the study shows that combining variable-centered and person-centered approaches can shed light on both general mechanisms and individual differences. Taken together, these findings suggest that routine monitoring of nurses’ work-related quality of life, along with interventions matched to their psychological capital profiles, may help sustain innovative capacity in high-strain clinical settings. Future research could extend these findings by examining the proposed pathways using longitudinal designs, exploring whether the psychological capital profiles identified in this study are replicable across other nursing populations, and investigating whether interventions targeting psychological capital are associated with sustained improvements in innovative behavior. Qualitative approaches could similarly enrich our understanding by capturing how emergency and critical care nurses experience and navigate the interplay of stress and innovation in their daily practice.

## Data Availability

The raw data supporting the conclusions of this article will be made available by the authors, without undue reservation.
